# Influence of Evaporation Drying on the Porous Properties of Carbon/Carbon Composite Xerogels

**DOI:** 10.3390/polym13162631

**Published:** 2021-08-07

**Authors:** Kriangsak Kraiwattanawong, Noriaki Sano, Hajime Tamon

**Affiliations:** 1 Department of Chemical Engineering, School of Engineering, King Mongkut’s Institute of Technology Ladkrabang, Bangkok 10520, Thailand; 2 Department of Chemical Engineering, Kyoto University, Kyoto 615-8510, Japan; sano@cheme.kyoto-u.ac.jp (N.S.); tamon@cheme.kyoto-u.ac.jp (H.T.)

**Keywords:** composite, evaporation drying, porous properties, carbon xerogel, nitrogen adsorption

## Abstract

Carbon/carbon (C/C) composite xerogels dried by evaporation were prepared in this study to observe the change of their porous properties and their morphology by nitrogen sorption apparatus and a scanning electron microscope. Resorcinol and formaldehyde (RF) sols as a matrix phase and cotton fibers (CF) as a dispersed phase were mixed and gelated to be CF/RF composite hydrogels. The composite hydrogels were exchanged by t-butanol (TBA), dried by evaporation at 50 °C, and carbonized at 1000 °C to become the C/C composite xerogels. The results show that the CF addition does not decrease the mesoporous properties of the C/C composite xerogels. Moreover, the CF addition can alleviate the pore shrinkage, and it can maintain the mesopore structure. The mesopore size and the micropore size of C/C composites are insignificantly changed because the CF addition and the solvent exchange using TBA may suppress the pore shrinkage despite the gas-liquid interface existing during the evaporation drying.

## 1. Introduction

Organic aerogels and carbon aerogels have a high BET surface area (*S*_BET_) and large mesopore volumes (*V*_mes_). They can be prepared by the sol-gel polymerization of resorcinol with formaldehyde (RF), followed by supercritical drying with carbon dioxide and carbonization under inert gas [1,2,3,4,5,6,7,8]. Their pore structure can be controlled by changing the amounts of reactants, catalysts, and water [6]. Therefore, they can be used in various applications: adsorbents, column packing materials for high-performance liquid chromatography, and electrode materials for electric double layer capacitors, and so forth. However, the supercritical drying is a sophisticated method, and the cost of supercritical equipment is expensive. RF carbon cryogels with excellent mesoporous properties are prepared by freeze drying with solvent exchange using t-butanol (TBA), and their porous properties can be controlled by the synthesis conditions [9,10,11,12]. Freeze drying is an inexpensive method, but it needs an expensive high vacuum pump and deep chiller.

Evaporation drying, a simple and very cheap method, has been used to prepare the porous carbon xerogels by Mayer et al. [13]. With the simple drying method, porous carbon xerogels have been investigated [14,15,16,17,18,19,20,21,22,23,24,25]. Many researchers have used solvent exchange in the porous development of carbon xerogels because solvent exchange can alleviate the capillary force from the gas-liquid interface, contributing to the mesoporous carbon xerogels [15,16,17,18,19,20,21,22,23,24]. Wu et al. studied the solvent species (methanol, ethanol, isopropanol) used as solvents in the preparation of alcogels [21]. Isopropanol resisted structural collapse under ambient pressure drying. Furthermore, Kraiwattanawong et al. compared the solvent species (water, ethanol, acetone, TBA, and toluene) for the preparation of carbon xerogels [25]. It was found that TBA was a suitable solvent for the preparation of mesoporous carbon xerogels because TBA gave low surface tension, decreasing the pore shrinkage under evaporation or vacuum drying.

Carbon/carbon (C/C) composites possess heterogeneous structures composed of a carbon matrix phase and a dispersed phase. Herein, the normal carbon cryogels prepared from the RF cryogels are applied as the carbon matrix phase, and the cotton fibers (CF) are used as the dispersed phase [26,27,28]. The dispersed phase can be changed by using various materials such as carbon cloth [29,30], activated carbon fibers [31], polymeric fibers [32], and cellulose [32]. The C/C composites are also studied for their mechanical properties [30,31], porous properties [26,27,28,31], electrochemical properties [28,33], and thermal insulation properties [34], and so forth. The C/C composites can allow us to design the appropriate properties to solve specific scientific and engineering problems. Evaporation drying is a convenient and cheap method, and CFs are renewable, natural, and very inexpensive materials in volume. The C/C composite xerogels are interesting for production on a commercial scale. Therefore, the aim of this work is to study the influence of evaporation drying on the mesoporosity and the microporosity of C/C composite xerogels. CFs are used as the dispersed phase and RF sol as the matrix phase. Nitrogen adsorption apparatus is used to estimate the porous properties of C/C composite xerogels. A scanning electron microscope (SEM) is used to investigate the mesopore structure of C/C composite xerogels.

## 2. Experimental

### 2.1. Synthesis of C/C Composites

The C/C composite xerogels can be synthesized by the sol-gel polymerization of an RF sol with CFs. The RF sol was prepared from resorcinol (Wako Pure Chemical Industries Inc.,Osaka, Japan, research grade, 99.7 wt%), formaldehyde (Wako Pure Chemical Industries Inc., research grade, 37 wt%, stabilized by 8 wt% methanol), sodium carbonate (Na_2_CO_3_) (C) (Nacalai Tesque Inc., Kyoto, Japan, research grade, 99.7%) as a basic catalyst, and distilled water (W) as a solvent. The mixture was gelated at 25 °C and cured at 25 °C for one day, at 50 °C for one day, and at 85 °C for three days. Note that the curing temperature of 85 °C for three days increases the stiffness of the CF/RF composites. The longer the curing time, the better the stiffness obtained. The stiffness affects the strength of CF/RF composites during drying and carbonization. If the pore structure of a CF/RF composite collapses during evaporation due to poor stiffness, the final product will have low porosity. Good stiffness of CF/RF composites is thus required. Later the composite wet gels were cut to a thin wafer (ca. 1 mm thickness). They were then exchanged with TBA (Wako Pure Chemical Industries Inc., research grade, 99 wt%). The fresh TBA was exchanged daily to remove water inside the CF/RF composite hydrogels [25]. The exchanged CF/RF composites were dried in the oven at 50 °C for three days. Note that the evaporation can remove TBA within one or two days based on the synthesis conditions. Three days are enough time to dry the CF/RF composites for every synthesis condition. Subsequently the CF/RF composite xerogels were carbonized in a quartz reactor inside the horizontal furnace to obtain the C/C composite xerogels [5]. Nitrogen flowed into the quartz reactor at 200 cm^3^/min. The temperature was ramped from room temperature to 250 °C at 250 °C/h, and it was maintained at 250 °C for 2 h. Next, the temperature was increased to 1000 °C at 250 °C/h, and it was kept at 1000 °C for 4 h. Under the carbonization step, CF is converted to carbon fibers (CaF). The C/C composite xerogels synthesized by the molar ratio of resorcinol to formaldehyde (R/F), the molar ratio of resorcinol to sodium carbonate (R/C), the mass concentration of resorcinol to distilled water (R/W), and the mass ratio of CF to RF (CF/RF) are summarized and named in Table 1. For the carbon cryogels, the exchanged CF/RF composites were dried by the freeze-drying technique [26,27,28] and carbonized in the same conditions as the C/C composite xerogels.

### 2.2. Characterization

The N_2_ adsorption and desorption isotherms were measured at −196 °C by the N_2_ adsorption apparatus (BEL Japan Inc., BELSORP-mini). *S*_BET_ was calculated by the N_2_ adsorption isotherm branch. The mesopore size distribution (MesPSD), the *V*_mes_ value, and the mesopore radius (*r*_p_) were calculated by the Dollimore–Heal method [35]. From the MesPSD curve, the mesopore radius at its highest peak (*r*_mes,peak_) was observed, representing as an average *r*_p_ value. The micropore volume (*V*_mic_) was estimated by the Dubinin–Radushkevich method [36]. The t-plot method [37] was utilized to estimate the micropore surface area (*S*_mic_). The HK method [38] was used to calculate the micropore diameter (*D*_p_) and the micropore size distribution (MicPSD). From the MicPSD curve, the micropore diameter at its highest peak (*D*_mic,peak_) was investigated and used as an average *D*_p_ value. The structure of the C/C composites was observed by field emission scanning electron microscope (JEOL, JSM-6700F). The SEM images were observed without platinum coverage because the C/C composites had electrical conductivity.

## 3. Results and Discussion

### 3.1. Morphology of C/C Composite Xerogel

**Figure 1:**
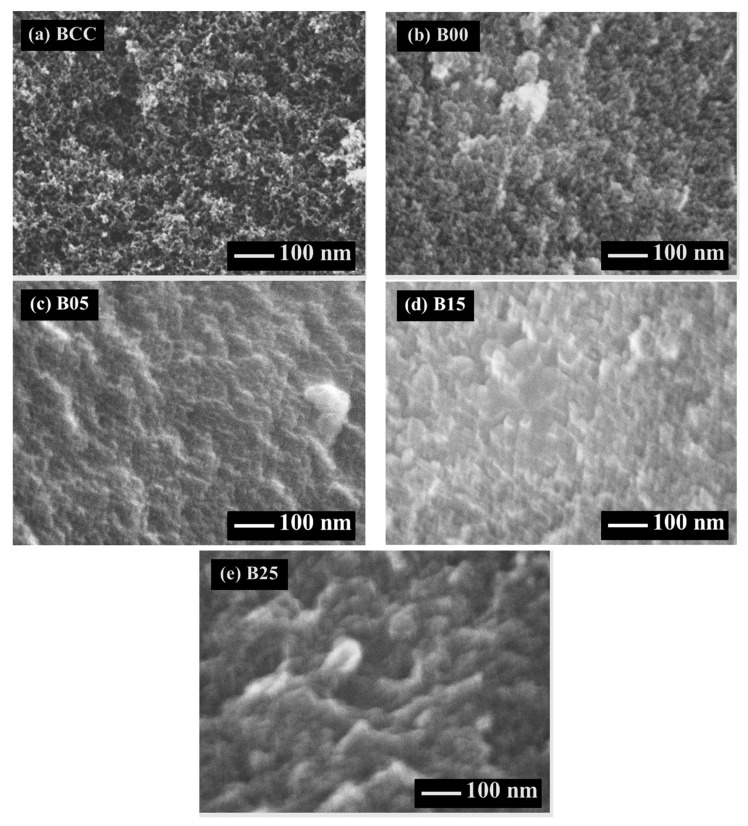
SEM images at 100,000× magnification of mesoporous carbon prepared at R/C = 200 mol/mol and R/W = 0.25 g/cm^3^: (**a**) carbon cryogel, (**b**) carbon xerogel, (**c**) C/C composite xerogel at CF/RF = 0.05 g/g, (**d**) C/C composite xerogel at CF/RF = 0.15 g/g, and (**e**) C/C composite xerogel at CF/RF = 0.25 g/g.

**Figure 2:**
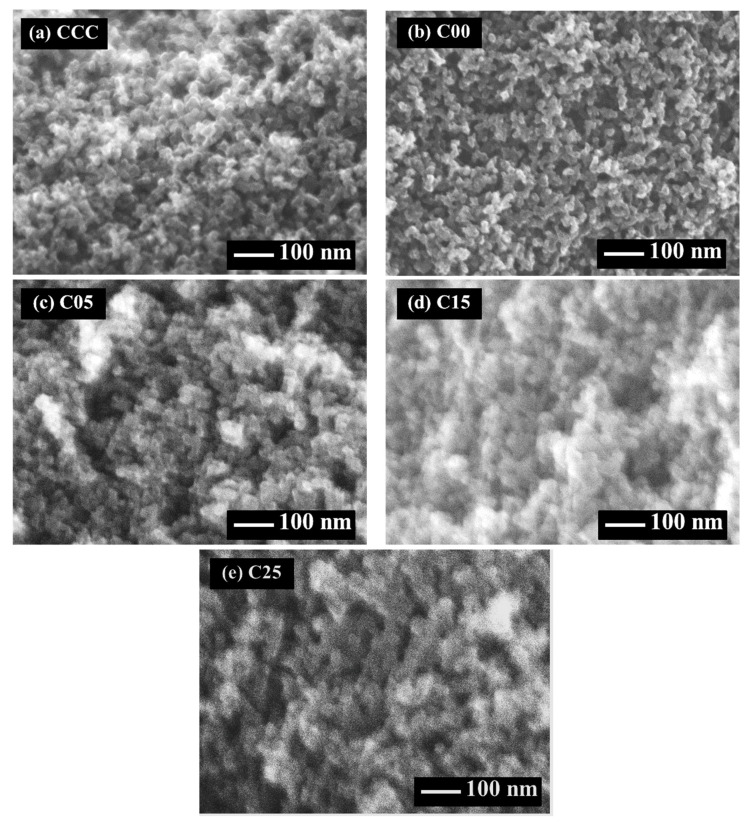
SEM images at 100,000× magnification of mesoporous carbon prepared at R/C = 500 mol/mol and R/W = 0.25 g/cm^3^: (**a**) carbon cryogel, (**b**) carbon xerogel, (**c**) C/C composite xerogel at CF/RF = 0.05 g/g, (**d**) C/C composite xerogel at CF/RF = 0.15 g/g, and (**e**) C/C composite xerogel at CF/RF = 0.25 g/g.

The microstructures of carbon cryogels, the carbon xerogels, and the C/C composite xerogels are observed, and the SEM images are presented in Figure 1 and Figure 2. The carbon structure of carbon cryogels, carbon xerogels, and the C/C composite xerogels consist of nanoparticles forming the mesopores. The carbon structure of carbon cryogels is similar to the carbon structure of carbon xerogels and C/C composite xerogels. Although freeze-drying can reduce the pore collapse by sublimation, evaporation drying can suppress pore shrinkage due to the vapor-liquid interface forming the capillary force. If the solvent (water) is not exchanged by TBA, water inside the tiny pores generates large surface tension during evaporation drying, ruining the pore network [25]. TBA has low surface tension, decreasing the capillary effect in the tiny pores [25]. When the evaporation is applied to the CF/RF composites, the gel network of CF/RF composites can resist the pore collapse as well. 

By using R/C = 500 mol/mol and R/W = 0.25 g/cm^3^, the carbon cryogel, the carbon xerogel, and the C/C composite xerogels have large nanoparticles, as shown in Figure 2, because the high R/C ratio can contribute such large nanoparticles [12]. The gel-network of carbon cryogels, the carbon xerogels, and the C/C composite xerogels are slightly shrunk by the evaporation. This result can be explained by the fact that the carbon gel preparation at a high R/C ratio gives low shrinkage of the microstructure [8]. Therefore, the SEM images can confirm that the preparation of C/C composite xerogels does not result in a change in their porous structure from the normal carbon xerogels at a high R/C ratio.

### 3.2. Porous Properties of C/C Composite Xerogels Dried by Evaporation Drying 

**Figure 3:**
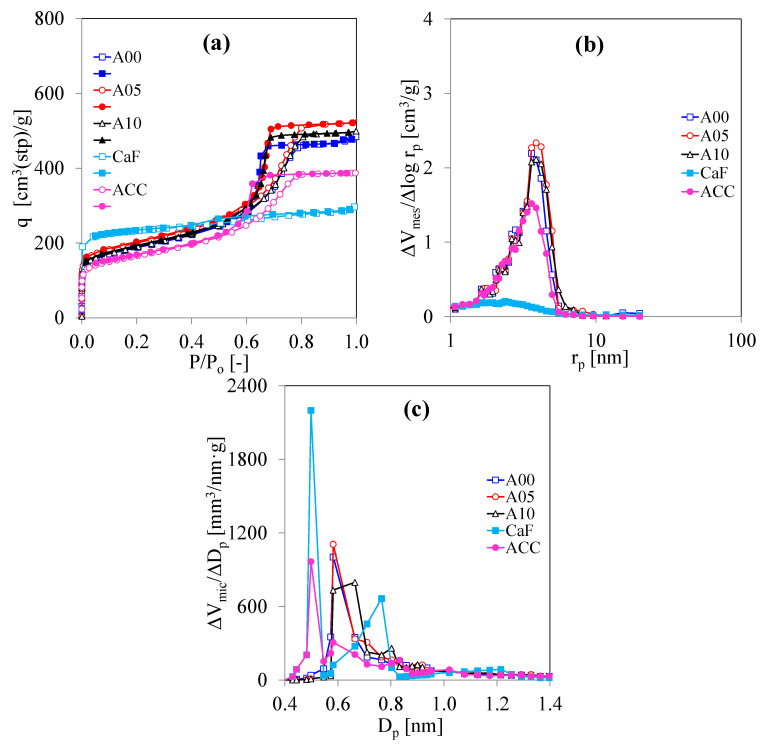
N_2_ adsorption analysis of CaF and C/C composite xerogels prepared by using the CF/RF ratio for 0.00–0.10 g/g at R/C = 200 mol/mol and R/W = 0.5 g/cm^3^: (**a**) N_2_ adsorption and desorption isotherms at −196 °C (open symbols = adsorption; closed symbols = desorption), (**b**) mesopore size distribution, and (**c**) micropore size distribution.

Figure 3 shows the N_2_ adsorption analysis of CaF, ACC, and C/C composite xerogels at −196 °C, consisting of the N_2_ adsorption and desorption isotherms, MesPSD, and MicPSD. In Figure 3a, the type-I isotherm of CaF suggests that CaF contains micropores. Besides, the small hysteresis loop of CaF hints that CaF has few mesopores. Contradictorily, Figure 3a also reveals the type-IV isotherms of C/C composites having micropores and mesopores similar to the type-IV isotherms of ACC. Herein, at the high relative pressure, the N_2_ amounts adsorbed on ACC are less than the adsorbed N_2_ amounts on A00. This result suggests that the porosity of ACC is less than the porosity of A00, but this consequence will be discussed later. Figure 3b demonstrates the MesPSD curve of the CaF and C/C composites estimated by using the DH method to the adsorption branch. The MesPSD curve of CaF confirms that CaF has few mesopores corresponding to its N_2_ adsorption isotherm. The MesPSD curve of ACC is identical to the MesPSD curve of C/C composite xerogels, but the MesPSD curve of ACC is the lowest. Among the C/C composite xerogels, MesPSD of A00 is similar to MesPSD of A05 and A10. Therefore, in these specimens, not only does the CF addition in the preparation of C/C composite xerogels maintain the pore network, but the CF addition also increases the mesoporosity with a low production cost. Figure 3c shows the MicPSD curve of the CaF and C/C composite xerogels calculated by using the HK method to the adsorption branch. The MicPSD curve of CF suggests a bimodal micropore structure, while the MicPSD curves of ACC and A00 indicate a uniform structure. When the C/C composite xerogels are prepared, the MicPSD curve of A00 is the same as the MicPSD curve of A05, while the MicPSD curve of A10 becomes broad.

**Figure 4:**
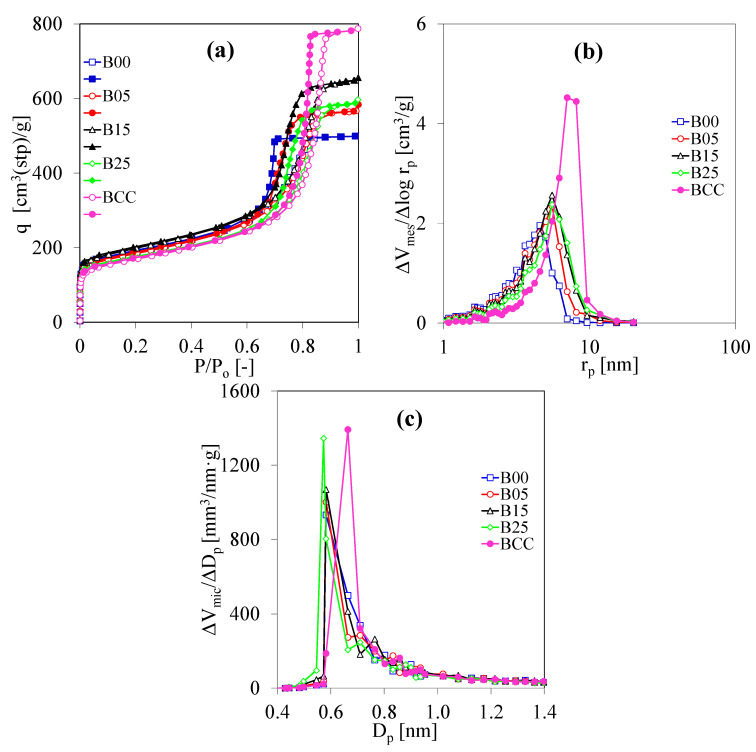
N_2_ adsorption analysis of CaF and C/C composite xerogels prepared by using the CF/RF ratio for 0.00–0.25 g/g at R/C = 200 mol/mol and R/W = 0.25 g/cm^3^: (**a**) N_2_ adsorption and desorption isotherms at −196 °C (open symbols = adsorption; closed symbols = desorption), (**b**) mesopore size distribution, and (**c**) micropore size distribution.

Figure 4 demonstrates the N_2_ adsorption analysis of the BCC and C/C composite xerogels. The amounts of N_2_ adsorbed on BCC at a high relative pressure is large, as shown in Figure 4a, whereas the amounts of N_2_ adsorbed on B00 are smaller. In this synthesis condition, freeze-drying can maintain the porosity of carbon gel better than evaporation drying. This may be because freeze-drying generally inhibits the microstructure collapse well, while evaporation drying cannot avoid the gas-liquid interface inducing pore shrinkage. This result is related to previous works [16,25]. By the CF addition for the preparation of C/C composite xerogels, the amounts of N_2_ adsorbed on B05, B15, and B25 are larger than the amounts of N_2_ adsorbed on B00; consequently, the porosity of B05, B15, and B25 are higher than the porosity of B00. These results imply that the CF addition helps the evaporation drying to increase the porous properties of C/C composite xerogels. Figure 4b shows that the MesPSD curve of BCC is uniform, but the MesPSD curves of C/C composites are rather broad with a smaller *r*_mes,peak_. This is because evaporation drying allows pore shrinkage due to the capillary force. However, the solvent exchange by TBA can decrease pore collapse [25]. For Figure 4c, although evaporation drying decreases the average micropore size of C/C composite xerogels to 0.573 nm and 0.582 nm, freeze-drying can contribute to a large average micropore size at 0.664 nm. This result is also influenced by the drying method.

**Figure 5:**
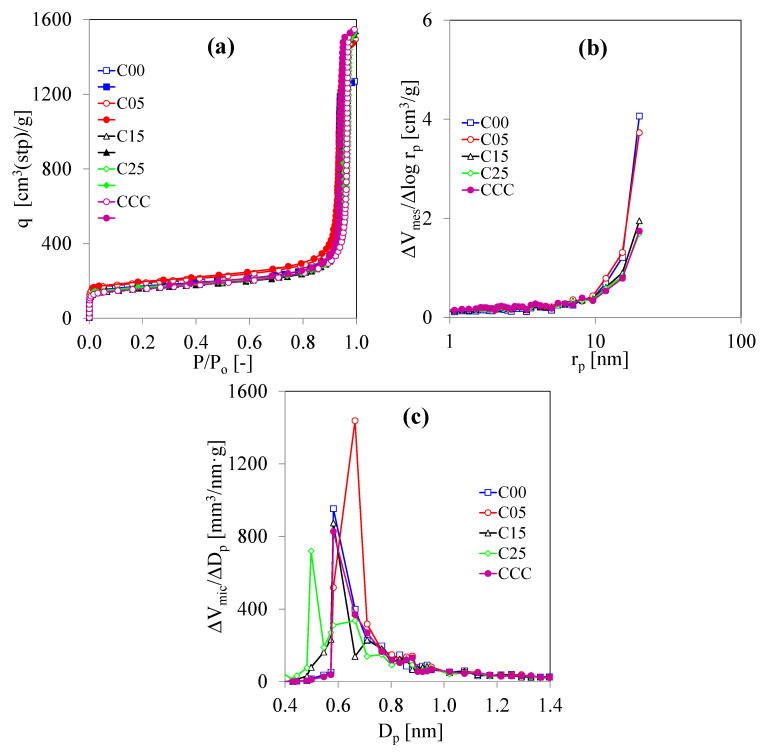
N_2_ adsorption analysis of CaF and C/C composite xerogels prepared by using the CF/RF ratio for 0.00–0.25 g/g at R/C = 500 mol/mol and R/W = 0.25 g/cm^3^: (**a**) N_2_ adsorption and desorption isotherms at −196 °C (open symbols = adsorption; closed symbols = desorption), (**b**) mesopore size distribution, and (**c**) micropore size distribution.

Figure 5a indicates the N_2_ adsorption and desorption isotherms on CCC and the C/C composite xerogels at −196 °C. The shape of every N_2_ adsorption isotherm is similar. Noticeably, the shape of the N_2_ adsorption isotherm is different from the previous synthesis conditions in Figure 3a and Figure 4a. The mesopores are formed by the void among the nanoparticles. The increase in R/C ratio increases the nanoparticle sizes, enlarging the mesopore radius [7,8]. At 500 mol/mol of the R/C ratio, the mesopore radius becomes large and macropores can be formed simultaneously. The hysteresis loops become narrow and steep with the larger amounts of N_2_ adsorbed at the high-pressure ratio related to the large mesopores and macropores, as shown in Figure 5a. On the other hand, the hysteresis loops in Figure 4a are large loops corresponding to the mesopore structure. Here, Figure 5b shows that the mesopores of every carbon specimen have a very large pore radius (*r*_p_ > 10 nm). Note that Figure 5b shows only half of the mesopore size distribution in the *r*_p_ term because the DH method demonstrates the mesopore range. Furthermore, Figure 5c shows that the MicPSD curves of CCC and C00 are identical.

Table 2 reports the porous properties of CaF, carbon cryogels, and C/C composite xerogels. The results show that BCC has a larger *V*_mes_ value than B00, but ACC and CCC have smaller *V*_mes_ values than A00 and C00, respectively. Generally, although freeze-drying is better than evaporation to dry the hydrogels, the synthesis conditions of hydrogels also play an important role equal to that of the drying method to determine the porosity of carbon products [16,20,24]. For the C/C composite xerogels, A00, A05, and A10 possess porous properties better than those of ACC dried by freeze-drying. Moreover, the *r*_mes,peak_ value of the C/C composite xerogels can be insignificantly changed from 3.6 nm to 3.9 nm. The results imply that evaporation drying with solvent exchange using TBA can be effectively applied to the preparation of the C/C composite xerogels with an insignificant change of *r*_mes,peak_ value. In other words, CF addition can keep the mesopore radius. For the micropore size, the *D*_mic,peak_ values of ACC and A00 are equal to the *D*_mic,peak_ value of A05 at 0.582 nm, but the *D*_mic,peak_ value of A10 is initially deviated to 0.664 nm. Nonetheless, the micropore size is not greatly changed.

For other synthesis conditions, however, evaporation drying with solvent exchange using TBA cannot prevent pore shrinkage. The *V*_mes_ value of B00 is smaller than the *V*_mes_ value of BCC by about 40%, and the mesopore size is also slightly decreased by pore shrinkage, from 6.2 nm to 4.6 nm. When the CF addition in the preparation of C/C composites is used, the *V*_mes_ value and the mesopore size of the C/C composite xerogels (B05, B15, and B25) increase to a greater extent than those of B00. Consequently, the mesopore shrinkage of C/C composite xerogels by evaporation drying can be alleviated by CF addition. Here, the *D*_mic,peak_ values are almost constant, as reported in Table 2. If the R/C ratio is increased to 500 mol/mol, C00 has a larger *V*_mes_ value than CCC. Nonetheless, the more CF addition herein seemingly decreases the *V*_mes_ value. It is found that the *V*_mic_ value is slightly increased by evaporation drying with solvent exchange using the application of TBA. The *D*_mic,peak_ value also slightly oscillates around 0.582 nm. Therefore, the preparation of C/C composite xerogels can maintain the mesopore size and micropore size nearly equivalent to the carbon cryogels at the same RF synthesis conditions. Compared with the carbon cryogel, previous work has illustrated that the *V*_mes_ value of a carbon xerogel was significantly decreased by evaporation drying, but the *V*_mic_ value could be maintained [25]. Therefore, the CF addition in the preparation of C/C composite xerogels can develop the mesopores and the micropores by evaporation drying with solvent exchange using TBA. 

## 4. Summary and Conclusions

The C/C composite xerogels were synthesized from the mixture of RF sol and CF with a slightly basic solution, gelated at 25 °C, cured at 85 °C, exchanged by TBA, dried by evaporation drying, and carbonized at 1000 °C. C/C composite xerogels were prepared by using three RF sols and several CF/RF ratios. The carbon cryogels dried by freeze-drying were also prepared to compare with the normal carbon xerogels. The porous properties and the pore structure of C/C composite xerogels were clarified by the N_2_ adsorption apparatus and scanning electron microscope, respectively. Their porous properties were calculated from the adsorption branch by the BET method, the DH method, the t-plot method, the HK method, and the DR method. The following conclusions were reached.

(a) The preparation of C/C composite xerogels does not result in a change in their carbon structure compared to the carbon xerogels and the carbon cryogels at a high R/C ratio.
(b) Not only does the CF addition develop the mesopores of C/C composite xerogels, but the CF addition also maintains the mesopore size and the micropore size to a level nearly equivalent to the carbon cryogels under the same RF synthesis conditions by evaporation drying with solvent exchange using TBA.
(c) The mesopore shrinkage of C/C composite xerogels during evaporation drying can be alleviated by CF addition.
(d) Evaporation drying with solvent exchange using TBA can prevent the decrease in micropore volume in the preparation of C/C composite xerogels.

## Figures and Tables

**Table 1:** Nomenclatures of C/C composite cryogels prepared under various synthesis conditions.

Symbol	CF/RF (g/g)	R/C (mol/mol)	R/W (g/cm^3^)	C/W (× 10^5^ mol/cm^3^)	Drying Method
A00	0.00	200	0.50	250	Evaporation
A05	0.05	200	0.50	250	Evaporation
A10	0.10	200	0.50	250	Evaporation
ACC	0.00	200	0.50	250	Freeze drying
B00	0.00	200	0.25	125	Evaporation
B05	0.05	200	0.25	125	Evaporation
B15	0.15	200	0.25	125	Evaporation
B25	0.25	200	0.25	125	Evaporation
BCC	0.00	200	0.25	125	Freeze drying
C00	0.00	500	0.25	50	Evaporation
C05	0.05	500	0.25	50	Evaporation
C15	0.15	500	0.25	50	Evaporation
C25	0.25	500	0.25	50	Evaporation
CCC	0.00	500	0.25	50	Freeze drying

**Table 2:** Porous properties of CaF and C/C composite xerogels prepared under various synthesis conditions.

Symbol	*S*_BET_ (m^2^/g)	*S*_mic_ (m^2^/g)	*V*_mes_ (cm^3^/g)	*V*_mic_ (cm^3^/g)	*r*_mes,peak_ (nm)	*D*_mic,peak_ (nm)
ACC	590	310	0.48	0.19	3.6	0.582
A00	680	360	0.60	0.22	3.6	0.582
A05	730	390	0.66	0.23	3.9	0.582
A10	680	340	0.63	0.22	3.9	0.664
BCC	700	350	1.04	0.22	6.2	0.664
B00	680	380	0.63	0.21	4.6	0.582
B05	660	360	0.74	0.21	5.5	0.582
B15	720	390	0.85	0.23	5.5	0.582
B25	620	340	0.78	0.21	5.5	0.573
CCC	590	440	0.58	0.19	nm	0.582
C00	630	500	0.87	0.21	>20.02	0.582
C05	720	560	0.88	0.23	>20.02	0.664
C15	590	450	0.62	0.19	>20.02	0.582
C25	620	490	0.57	0.20	>20.02	0.498
CaF	910	890	0.13	0.28	2.5	0.498

## Data Availability

The data presented in this study are available on request from the corresponding author.
